# Smoking and colorectal cancer in inflammatory bowel disease: Quantity matters?

**DOI:** 10.1002/ueg2.12433

**Published:** 2023-06-30

**Authors:** Irene Marafini, Giovanni Monteleone

**Affiliations:** ^1^ Gastroenterology Unit Policlinico Universitario Tor Vergata Rome Italy; ^2^ Department of Systems Medicine University of “Tor Vergata” Rome Italy

Long‐standing colonic Crohn's disease (CD) and ulcerative colitis (UC) can be complicated by the development of colorectal cancer (CRC),^1^ the major contributor to IBD‐associated mortality. IBD‐associated colorectal neoplasia (CRN) is the culmination of multiple oncogenic molecular events, which occur in the setting of chronic inflammation and manifest microscopically with progressive morphological changes of the colon epithelium, a process indicated by the term “inflammation‐dysplasia‐cancer” sequence.^2^, ^3^, ^4^ Therefore, factors that either attenuate or enhance the ongoing colonic inflammation could potentially inhibit or increase the risk of CNR. For instance, it has been proposed that early and intensive therapy with biologics can favor mucosal healing, thereby preventing the ultimate development of CNR in high‐risk IBD patients.^5^, ^6^

In this issue of United European Gastroenterology Journal, Wijnands and co‐workers take on the important task to try to elucidate the association between cigarette smoking and the development of CRN in IBD patients, including possible dose effects.^7^ This is an interesting topic as smoking is known to positively influence the risk of many malignancies and worsen the course in CD, while it is protective in UC.^8^

The authors performed a prospective and multicentre study in 4 academic centers in the Netherlands and enrolled 575 IBD patients, with a balanced distribution between UC and CD, of which 501 were followed up. At the study index evaluation, 48% of patients were current or former smokers. The study outcome was the occurrence of recurrent CRN events, which included indefinite dysplasia, low‐grade dysplasia, high‐grade dysplasia (HGD), or CRC. Patients with prior HGD and CRC were excluded from the analysis.

CRN occurred in approximately 20% of patients with 1 case of HGD and 6 cases of CRC. In both the univariable and multivariate analyses, smoking status alone (ever vs. never) did not influence the development of recurrent CRN, whereas an increase in pack‐years was associated with a higher risk of recurrent CRN in patients with IBD. The analysis of pack years is a useful tool that takes into consideration the smoking intensity (dose per day) and the duration of the smoking habit. The formula consists of multiplying the average amount of cigarettes/day by the number of years of active smoking divided by 20 (contents of a pack of cigarettes) and is considered a more reliable measure of the smoker's lifetime tobacco burden.^9^ Surprisingly, the dose relationship between smoking and CNR was not significant when the two IBDs were considered separately, probably reflecting the small group of patients with UC and CD enrolled. The association between the pack years and the risk of recurrent CRN in the whole IBD group remained significant even after adjustment for mean endoscopic inflammation scores, which we can assume to be an indirect measure of the global inflammatory burden.^10^ However, more than one‐third of the endoscopic procedures were not considered adequate, raising the possibility that some dysplastic lesions have been missed. It is also noteworthy that of 6 patients diagnosed with CRC, 4 had never smoked, indicating that factors other than smoking contribute to the development of advanced CNR. Because only half of the patients had extensive colitis, and no information was given about the location of CRN, it remains conceivable that some neoplastic lesions described in the present study may be, indeed, sporadic and not IBD‐associated, in line with results of previous studies in the general population documenting an association of smoking pack years with sporadic adenoma and CRC.^11^, ^12^ Another issue that remains to be clarified is the impact of smoking on the development of non‐conventional dysplastic lesions in IBD, as these lesions are common in IBD patients and are the most common precursor lesions associated with the development of low‐grade tubuloglandular and mucinous adenocarcinomas.^13^, ^14^

Clinicians suggest CD patients stop their smoking habit, while there are more hesitations in giving the same recommendation to patients with UC, considering the protective role of smoking in UC.^8^ Data from the present study indicate that cigarette smoking cessation should be advised in all IBD patients to reduce their risk of CRN. This study provides a solid starting point for further work on this topic that should take into account other factors (e.g. alcohol intake, diet, concomitant therapy with chemo‐preventive drugs), which could influence the risk of IBD‐associated CNR.

## CONFLICT OF INTEREST STATEMENT

IM served as a speaker for Janssen and as a consultant for Galapagos; GM served as a consultant for First Wave BioPharma and filed a patent related to the treatment of inflammatory bowel diseases with Smad7 antisense oligonucleotides.

## Data Availability

Data sharing not applicable—no new data generated or the article describes entirely theoretical research.
